# Supplementary material for: A method for the rational selection of drug repurposing candidates from multimodal knowledge harmonization
Source: Sci Rep. 2021 May 26;11:11049. doi: 10.1038/s41598-021-90296-2 (PMC8155020; doi:10.1038/s41598-021-90296-2)
Supplement: Supplementary file 1 — Supplementary Figure 1. [file 41598_2021_90296_MOESM1_ESM.pdf]

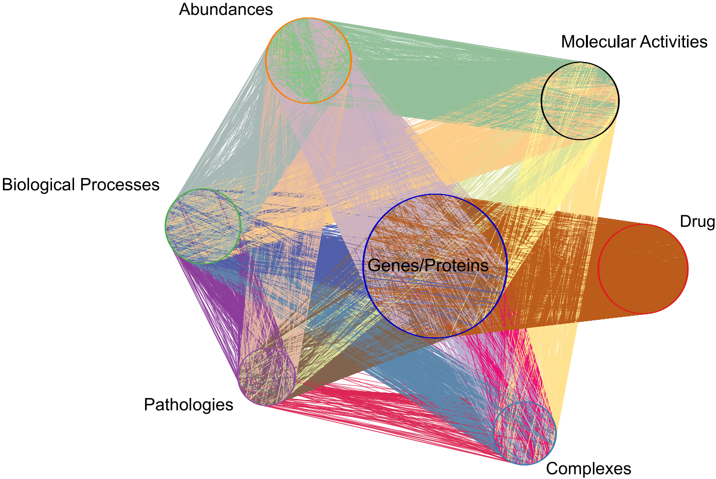
Abundances
Molecular Activities
Biological Processes
Drugs
Genes/Proteins
Pathologies
Complexes
