# Supplementary figures and images for: A method for the rational selection of drug repurposing candidates from multimodal knowledge harmonization
Source: Sci Rep. 2021 May 26;11:11049. doi: 10.1038/s41598-021-90296-2 (PMC8155020; doi:10.1038/s41598-021-90296-2)

a)

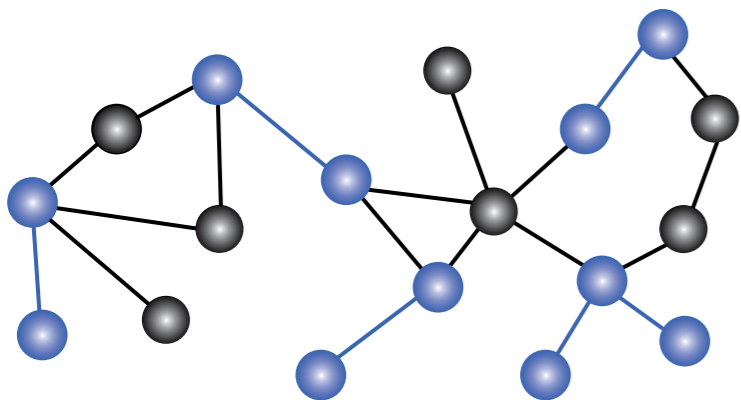

b)

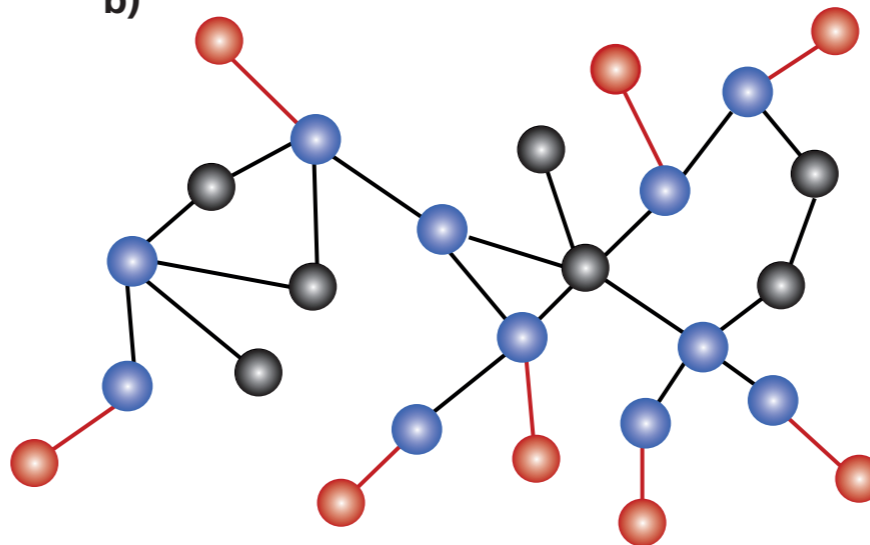

c)

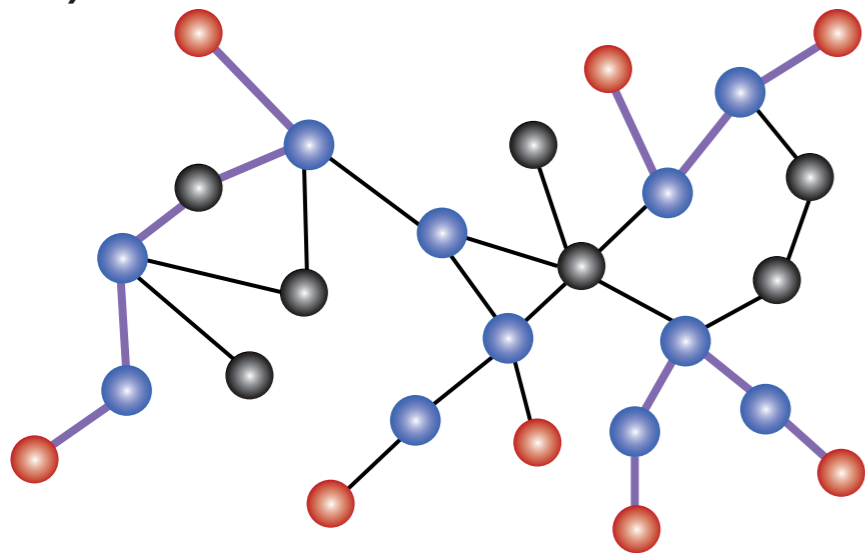

d)

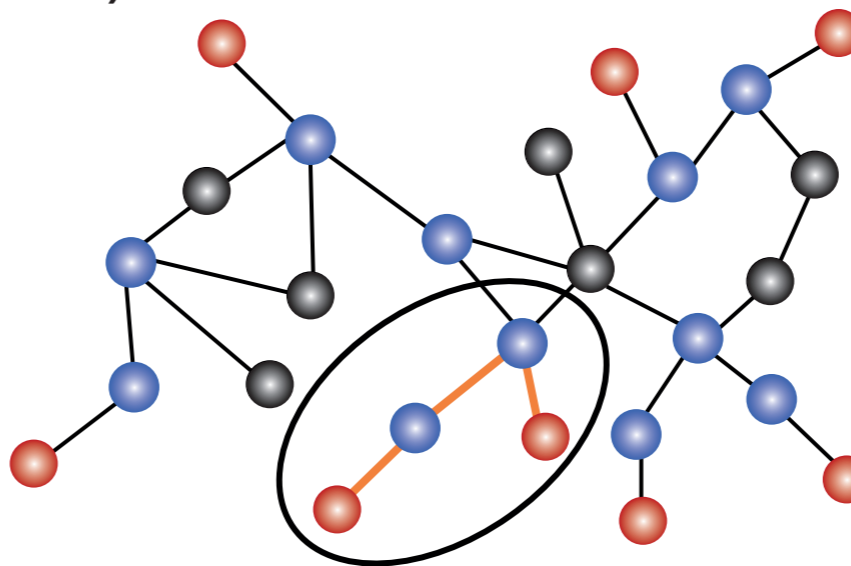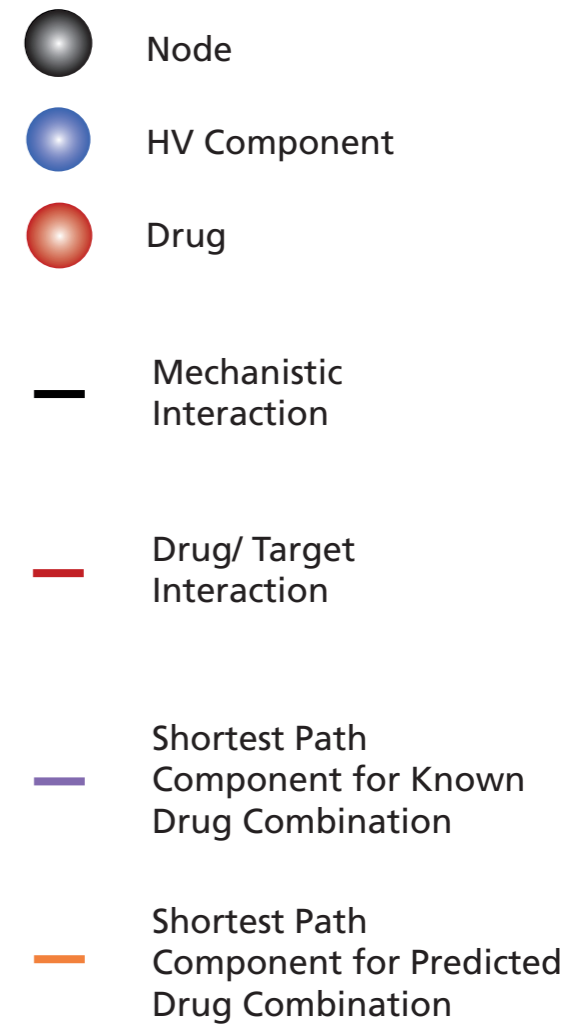

Supplement: Supplementary file 2 — Supplementary Figure 2. [file 41598_2021_90296_MOESM2_ESM.pdf]
